# New Gene Markers of Exosomal Regulation Are Involved in Porcine Granulosa Cell Adhesion, Migration, and Proliferation

**DOI:** 10.3390/ijms241411873

**Published:** 2023-07-24

**Authors:** Jakub Kulus, Wiesława Kranc, Magdalena Kulus, Dorota Bukowska, Hanna Piotrowska-Kempisty, Paul Mozdziak, Bartosz Kempisty, Paweł Antosik

**Affiliations:** 1 Department of Diagnostics and Clinical Sciences, Institute of Veterinary Medicine, Nicolaus Copernicus University in Torun, 87-100 Torun, Poland; jakub.kulus@umk.pl (J.K.); dbukowska@umk.pl (D.B.); 2 Department of Anatomy, Poznan University of Medical Sciences, 61-701 Poznan, Poland; wkranc@ump.edu.pl; 3 Department of Veterinary Surgery, Institute of Veterinary Medicine, Nicolaus Copernicus University in Torun, 87-100 Torun, Poland; magdalena.kulus@umk.pl (M.K.); pantosik@umk.pl (P.A.); 4 Department of Toxicology, Poznan University of Medical Sciences, 61-701 Poznan, Poland; hpiotrow@ump.edu.pl; 5 Department of Basic and Preclinical Sciences, Institute of Veterinary Medicine, Nicolaus Copernicus University in Torun, 87-100 Torun, Poland; 6 Physiology Graduate Faculty, College of Agriculture and Life Sciences, North Carolina State University, Raleigh, NC 27695, USA; pemozdzi@ncsu.edu; 7 Division of Anatomy, Department of Human Morphology and Embryology, Wroclaw Medical University, 50-367 Wroclaw, Poland; 8 Center of Assisted Reproduction, Department of Obstetrics and Gynecology, University Hospital and Masaryk University, 601 77 Brno, Czech Republic

**Keywords:** porcine granulosa cells, cellular signaling, extracellular vesicles, cell adhesion, cell migration and proliferation, transcriptomics, extracellular matrix

## Abstract

Exosomal regulation is intimately involved in key cellular processes, such as migration, proliferation, and adhesion. By participating in the regulation of basic mechanisms, extracellular vesicles are important in intercellular signaling and the functioning of the mammalian reproductive system. The complexity of intercellular interactions in the ovarian follicle is also based on multilevel intercellular signaling, including the mechanisms involving cadherins, integrins, and the extracellular matrix. The processes in the ovary leading to the formation of a fertilization-ready oocyte are extremely complex at the molecular level and depend on the oocyte’s ongoing relationship with granulosa cells. An analysis of gene expression from material obtained from a primary in vitro culture of porcine granulosa cells was employed using microarray technology. Genes with the highest expression (LIPG, HSD3B1, CLIP4, LOX, ANKRD1, FMOD, SHAS2, TAGLN, ITGA8, MXRA5, and NEXN) and the lowest expression levels (DAPL1, HSD17B1, SNX31, FST, NEBL, CXCL10, RGS2, MAL2, IHH, and TRIB2) were selected for further analysis. The gene expression results obtained from the microarrays were validated using quantitative RT-qPCR. Exosomes may play important roles regarding intercellular signaling between granulosa cells. Therefore, exosomes may have significant applications in regenerative medicine, targeted therapy, and assisted reproduction technologies.

## 1. Introduction

In oocyte maturation during oogenesis, granulosa cells (GCs) are necessary and surround the oocyte, interacting with it in numerous ways [1]. Within the granulosa cells found in the ovarian follicle, there are mural granulosa cells (mGCs), which occur at the periphery of the ovarian follicle and are closely associated with steroidogenesis and ovulation [2]. The second group of granulosa cells—namely, cumulus cells (CCs)—are those that are in direct contact with and surround the oocyte, forming close multiple intercellular connections with the oocyte [2]. These connections are of the gap junction type (nexus type), allowing, among other things, ion exchange [3]. Granulosa cells are responsible for the maturation of the oocyte, although they are also responsible for meiotic arrest through the regulation of cAMP levels. [4]. The adequate pool of these cells in the ovarian follicle depends on their division and proliferation. The proliferation of granulosa cells depends on a number of factors that are involved in the activation of signaling pathways, e.g., EGFR, PDGF, VEGF, TGF-β, MAPK, FAK/AKT, and ERK. Some of these factors have been well-known for years [5], but with active research, newer ones are being described, such as Procr (Protein C receptor) [6], Protegrin-1 [7], and the KAT2B gene [8]. The current direction of research should focus on a multifaceted view of cell signaling and its effects on the proliferation and migration of GCs. Moreover, properly functioning granulosa cells require efficient intercellular signaling, which involves the integrins, cadherins, and the extracellular matrix (ECM) that constitute the microenvironment for them [9]. The present study shows the upregulation of ontology groups of genes related to the effects of integrins on cell adhesion and the activation of signaling pathways. Integrins affect signaling dictated by Rho GTPase, which is involved in cytokinesis and cell migration [10]. In addition, the cytoskeleton, which is a dynamic structure in terms of composition and structure, also significantly affects intercellular signaling [11]. The GTPase RhoA is involved in the process of cytoskeleton change [12]. 

The upregulation of the vesicle-mediated transport ontology group demonstrated in this article suggests an important role for extracellular vesicles (EVs) in the activity of granulosa cells, especially in intercellular signaling. EVs are structures with a lipid membrane released outside the cell. They provide a carrier for proteins, RNAs, mRNAs, and microRNAs while being heavily involved in cell signaling. They are formed by budding or intracellular endocytic trafficking [13]. The cytoskeleton is also involved in EV secretion, which requires polymerization of actin located under the cytoplasmic membrane, allowing budding and the release of vesicles outside the cell [13]. Exosomes, belonging to extracellular vesicles, take direct and indirect roles in intercellular signaling and have been shown to play an important role in the functioning of the reproductive system [14,15,16,17]. These nanoparticles, through their involvement in the regulation of cell morphology, can promote cell adhesion [18]. In addition, exosomes transporting protein molecules released from cells have been suggested to promote cell migration, as described in inflammatory processes [19]. Recent reports have also indicated that exosomes are actively involved in the processes of proliferation [20] and the response to hypoxia [21]. Exosomes affect the composition of the ECM (through its remodeling), but, at the same time, the ECM affects the release of exosomes from the cell [22]. The ECM’s and exosomes’ formation and composition are important for the signaling pathways that take place in them, the passage of nutrients and hormones, and the initiation of many cellular mechanisms (migration and cell division) [23,24]. 

The success of the cell cycle requires interaction in multiple fields, both in the intracellular and extracellular environment. For this purpose, it is necessary to maintain proper interactions between the cytoskeleton and the extracellular matrix while varying cell adhesion and proliferation [25]. Microtubules are involved in the formation of the karyokinetic spindle [26], while intermediate filaments show an important role in cell adhesion and interaction with other components of the cytoskeleton [27]. The link between the cell cycle and cell adhesion has been confirmed through integrin receptors, which, by connecting the cell to the ECM, lead to the activation of a cell cycle signaling pathway progression, particularly the G1/S phase transition [28]. 

The interaction of the ECM, the cytoskeleton, and the release of EVs during the cell cycle affects the proper functioning of cells in terms of cell signaling, adhesion, proliferation, migration, and division [9,10,11,19,20,23,24,25,29]. These interactions on a molecular basis within GCs are not very well understood. Therefore, the goal of the current study was to investigate the expression profile of genes involved in the regulation of cell adhesion, migration, proliferation, and wound healing in porcine granulosa cells, as these are processes associated with exosomes’ formation and composition.

## 2. Results

**Figure 1:**
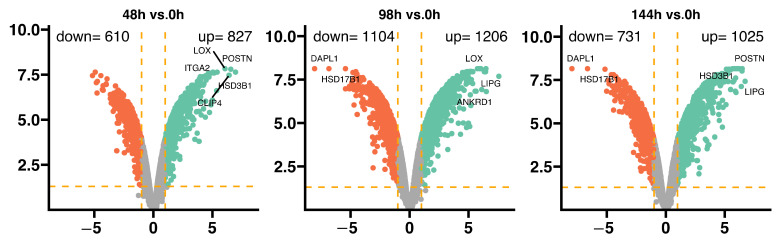
General expression profiles visualized as volcano plots. Each dot represents the mean expression (two biological replicates) of an individual gene obtained from a normalized microarray study. The orange dotted lines (cut-off values) were established according to the following parameters: |fold change| = 2 and *p* value = 0.05. Genes above the cut-off lines were considered as differentially expressed genes and are shown as red (downregulated) and green (upregulated) dots. The total numbers of upregulated and downregulated genes are given in the top right and top left corners, respectively. The symbols of the five most differentially expressed genes from each comparison are marked on the plots.

The porcine granulosa cells were collected at four time points, representing different stages of a short-term in vitro culture: 0 h (serving as an ex vivo reference); 48 h (representing the initial in-vitro-associated changes in culture); 96 h (an assumed “point of loss” of most of the cell’s physiological properties); and 144 h (the end point of the short-term culture). Obtaining information on the level and direction of gene expression in culture provides important new data regarding dynamic changes in the cell population. The transcriptomic profile of gene expression was compared to the control group (0 h). The general profile of the transcriptome changes is shown in Figure 1, where dots represent the mean gene expression. With respect to the assumed cut-off criteria for differentially expressed genes (|fold change| = 2, and *p* value < 0.05), we demonstrated 610 upregulated and 827 downregulated genes in the 48 h vs. 0 h comparison, 1104 upregulated and 1206 downregulated genes in the 96 h vs. 0 h comparison, and 731 upregulated and 1025 downregulated genes in the 144 vs. 0 h comparison. In the 48 h vs. 0 h comparison, the genes with the highest fold change of expression included: LOX, POSTN, ITGA2, HSD3B1, and CLIP4. In the 96 h vs. 0 h comparison, the most downregulated genes were DAPL1 and HSD17B1, with overexpression of LOX, LIPG, and ANKRD1 genes. In the 144 h vs. 0 h comparison, we observed decreased expression of DAPL1 and HSD17B1 and increased expression of POSTN, HSD3B1, and LIPG genes. 

**Figure 2:**
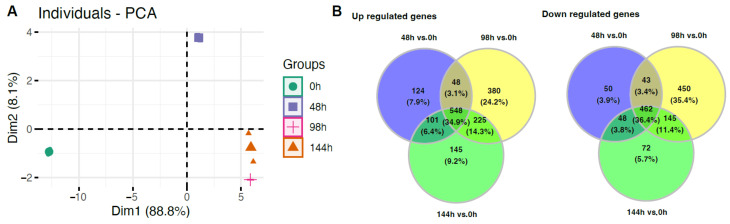
(**A**) Principal component analysis (PCA) plot of the first two components of the filtered microarray data set. (**B**) Venn diagrams indicating common upregulated and downregulated genes in all analyzed groups.

A principal component analysis (PCA) was performed to show similarities and differences in the analyzed transcriptomic profiles of the studied groups (Figure 2). PCA analysis showed a very strong separation of the studied groups, where the first component (Dim1) explained 88.8% of the differences between the groups. The 0 h and 48 h groups were considerably separated from the others, while the 96 h and 144 h groups were distinctly separate. The Venn diagram illustrates that many genes overlapped between the compared experimental conditions, and 548 genes were upregulated and 462 were downregulated in comparison to the control group, regardless of the duration of the cultivation. 

**Figure 3:**
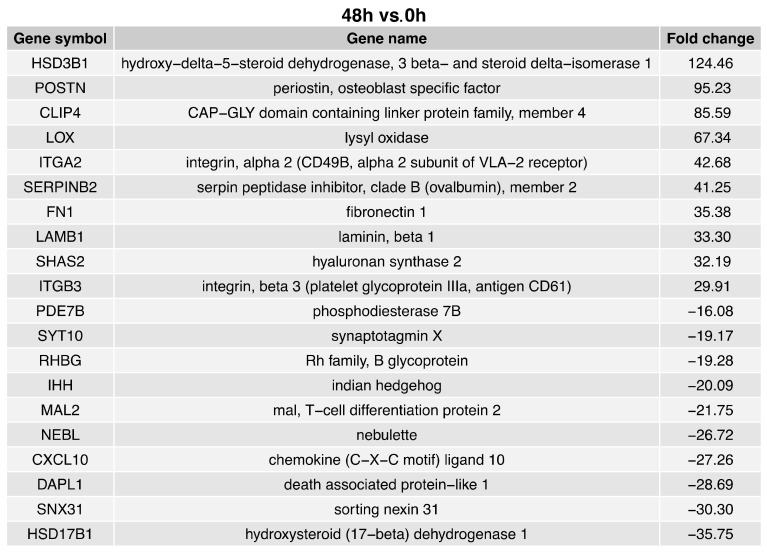
List of the top 20 genes with the highest (10 genes) and lowest (10) expression fold change between 48 h and 0 h of the cells’ cultivation.

The fold change values of the top ten upregulated genes in the 48 h vs. 0 h comparison (Figure 3) ranged from 124.46 to 29.91. The fold change values of the top ten downregulated genes in the 48 h vs. 0 h comparison ranged from −16.08 to −35.75. The ten genes with enhanced expression in the 48 h vs. 0 h comparison were: hydroxy-delta-5-steroid dehydrogenase 3-beta and steroid delta-isomerase 1 (HSD3B1); periostin (POSTN); CAP-GLY domain containing linker protein family member 4 (CLIP4); Lysol oxidase (LOX); integrin alpha 2 (ITGA2); serpin protease inhibitor clade B (ovalbumin) member 2 (SERPINB2); fibronectin 1 (FN1); laminin beta 1 (LAMB1); hyaluronian synthase 2 (SHAS2); and integrin beta 3 (ITGB3). The ten downregulated genes in the axis cells compared to the controls were: phosphodiesterase 7B (PDE7B); synaptotagmin X (SYT10); Rh family B glycoprotein (RHBG); Indian hedgehog (IHH); mal T-cell differentiation protein 2 (MAL2); nebulette (NEBL); chemokine (C-X-C motif) ligand 10 (CXCL10); death associated protein-like 1 (DAPL1); sorting nexin 31 (SNX31); and hydroxysteroid (17-beta) dehydrogenase 1 (HSD17B1). 

**Figure 4:**
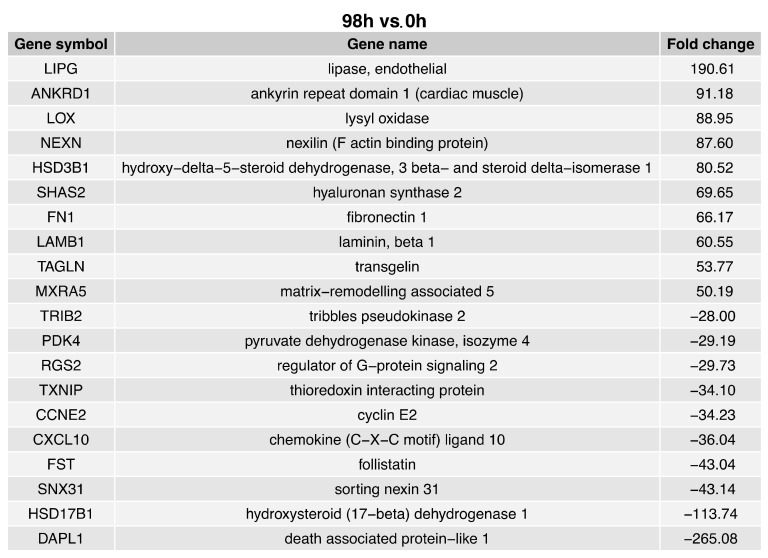
List of the top 20 genes with the highest (10 genes) and lowest (10) expression fold change between 96 h and 0 h of the cells’ cultivation.

The fold change values of the top ten upregulated genes in the 96 h vs. 0 h comparison (Figure 4) ranged from 190.61 to 50.19. The fold change values of the top ten downregulated genes in the 96 h vs. 0 h comparison ranged from −28.00 to −265.08. The ten genes with enhanced expression in the 96 h vs. 0 h comparison were: Lipase (LIPG); ankyrin repeat domain 1 (ANKRD1); lysyl oxidase (LOX); nexin (NEXN); hydroxy-delta-5−steroid dehydrogenase 3-beta and steroid delta-isomerase 1 (HSD3B1); hyaluronian synthase 2 (SHAS2); fibronectin 1 (FN1); laminin beta 1 (LAMB1); transgelin (TAGLN); and matrix-remodelling associated 5 (MXRA5). The ten downregulated genes in the 96 h vs. 0 h comparison were: Tribbles pseudokinase 2 (TRIB2); pyruvate dehydrogenase kinase isozyme 4 (PDK4); regulator of G-protein signaling 2 (RGS2); thioredoxin interacting protein (TXNIP); cyclin E2 (CCNE2); chemokine (C-X-C motif) ligand 10 (CXCL10); follistatin (FST); sortin nexin 31 (SNX31); hydroxysteroid (17-beta) dehydrogenase 1 (HSD17B1); and death associated protein-like 1 (DAPL1). 

**Figure 5:**
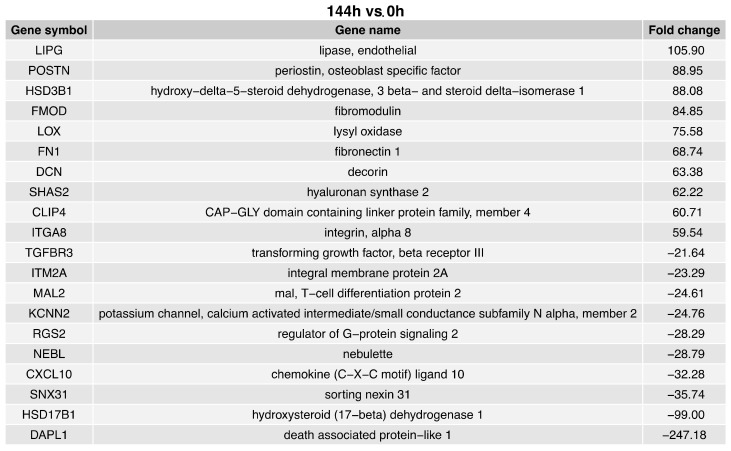
List of the top 20 genes with the highest (10 genes) and lowest (10) expression fold change between 144 h and 0 h of the cells’ cultivation.

The fold change values of the top ten upregulated genes in the 144 h vs. 0 h comparison (Figure 5) ranged from 105.90 to 59.54. The fold change values of the top ten downregulated genes in the 144 h vs. 0 h comparison ranged from −21.64 to −247.18. The ten genes with overexpression in the 144 h vs. 0 h comparison were: Lipase (LIPG); periostin (POSTN); hydroxy-delta-5-steroid dehydrogenase 3−beta and steroid delta-isomerase 1 (HSD3B1); fibromodulin (FMOD); lysyl oxidase (LOX); fibronectin 1 (FN1); decorin (DCN); hyaluronian synthase 2 (SHAS2); CAP-GLY domain containing linker protein family member 4 (CLIP4); and integrin alpha 8 (ITGA8).

The ten downregulated genes in the 144 h vs. 0 h comparison were: Transforming growth factor beta receptor III (TGFBR3); integral membrane protein 2A (ITM2A); mal T-cell differentiation protein 2 (MAL2); potassium channel, calcium activated intermediate/small conductance subfamily N alpha member 2 (KCNN2); regulator of G−protein signaling 2 (RGS2); nebulette (NEBL); chemokine (C-X-C motif) ligand 10 (CXCL10); sorting nexin 21 (SNX31); hydroxysteroid (17-beta) dehydrogenase 1 (HSD17B1); and death associated protein-like 1 (DAPL1). 

In conclusion, commonly overexpressed genes for all the analyzed groups were: HSD3B1, LOX, FN1, and SHAS2. Meanwhile, inhibited expression was observed in all groups for CXCL10, DAPL1, and SNX31 in comparison to the control. 

**Figure 6:**
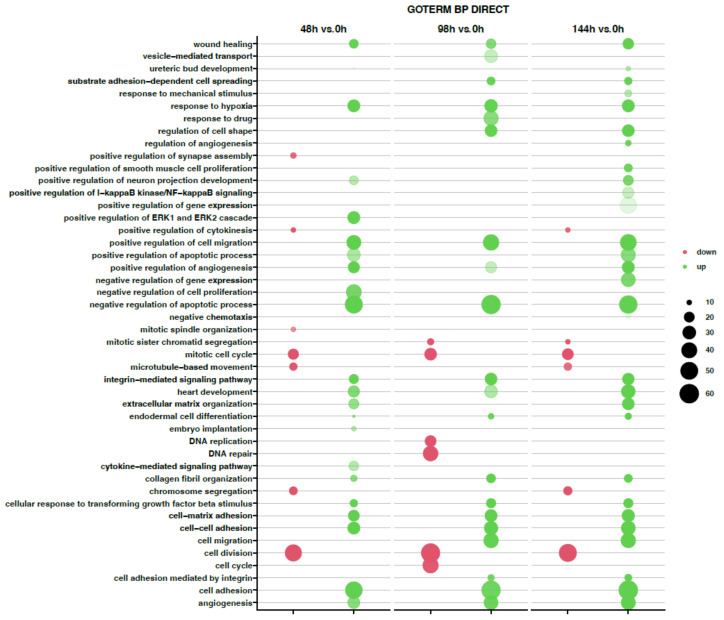
Bubble plot of overrepresented gene sets in DAVID GO PB DIRECT annotations database obtained from comparisons in gene expression profiles between 48 h, 96 h, and 144 h vs. control (0 h). The graph shows only the GO groups above the established cut-off criteria (p with correction < 0.05, a minimal number of genes per group >2). The size of each bubble reflects the number of differentially expressed genes assigned to the GO BP terms. The intensity of the bubble’s transparency displays a *p*-value (more transparent indicates closer to the *p* = 0.05 cut-off value). The green bubbles indicate overexpressed genes, and the red bubbles indicate downregulated genes.

Further analysis of the enrichment in the relevant ontological groups was performed using the Database for Annotation, Visualization, and Integrated Discovery (DAVID) bioinformatics tool with the GO BP Direct database (Figure 6). 

The analysis revealed 46 ontological groups. For all analyzed groups, some similarities in patterns in the gene expression profile were revealed between groups in comparison to the control. The downregulated genes were responsible for inhibition processes, such as cell division, mitotic cell cycle, and mitotic sister chromatid segregation. Meanwhile, upregulated genes comparable in all three groups were angiogenesis, cell adhesion, cell-cell adhesion, cell-matrix adhesion, cellular response to transforming growth factor beta stimulus, collagen fibril organization, endodermal cell differentiation, heart development, integrin-mediated signaling pathway, negative regulation of apoptotic process, positive regulation of angiogenesis, positive regulation of cell migration, response to hypoxia, and wound healing.

The relevant GO ontological groups with adjusted *p*-values below 0.05 and N per group > 2 are presented as a bubble in Figure 6. The analysis of the expression patterns in the 48 h group in comparison to the control revealed a total of twenty-two upregulated and seven downregulated GO BP terms. Meanwhile, in the 96 h group, we showed that twenty GO BP terms were activated and six terms were inhibited. The highest number of activated GO BP terms (29 terms) was observed at 144 h, with six inhibited terms. 

**Figure 7:**
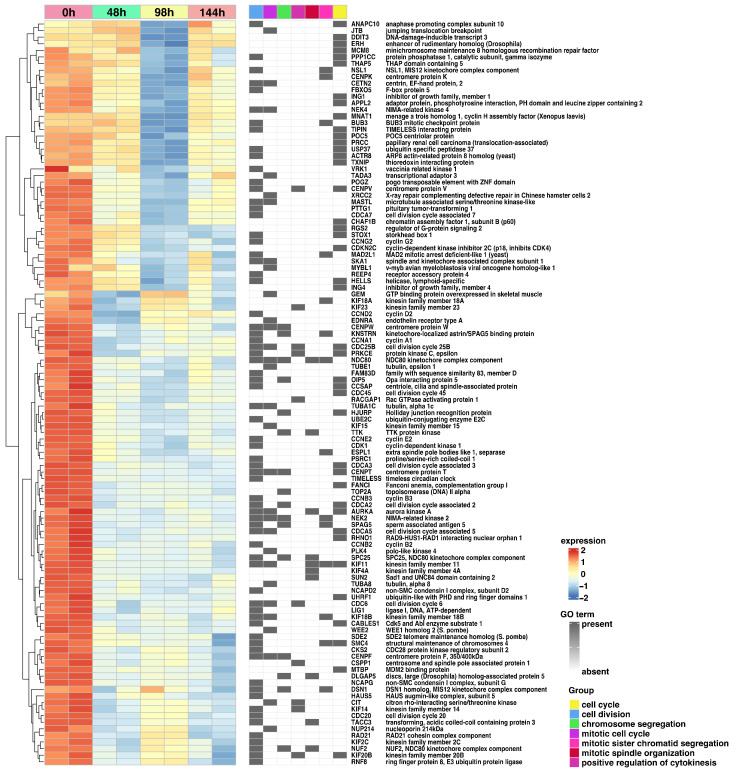
Heatmap with hierarchic clustering of differentially expressed genes in all analyzed groups. Genes belonging to the first seven most significantly enriched ontological groups (lowest adjusted *p*-value) are shown as dark squares. Expression values are scaled by rows and presented as colors and range from red (high expression) to yellow (moderate) to blue (low expression).

Hierarchic clustering of differentially expressed genes in all analyzed groups has been shown as a heatmap and presented in Figure 7. Genes belonging to the first seven most significantly enriched ontological groups (lowest adjusted *p*-value) are shown as dark squares. Expression values are scaled by rows and presented as colors and ranges. As observed, the expression of all analyzed genes decreased according to the time of the experiments. In accordance with previous results, most genes, regardless of the time of the experiment, were assigned to the cell division and cell cycle GO terms. 

Next, powerful bioinformatic tools, such as the Gene Set Enrichment Analysis (GSEA), were used to confirm the obtained results. The GSEA was performed for the 48/0 h, 96/0 h, and 144/0 h experimental groups. The normalized expression level data from the microarray were uploaded to the software and allowed us to generate the list of significantly represented terms from the Hallmark database software version 4.1.2 (BioConductor software, Boston, MA, USA). 

**Figure 8:**
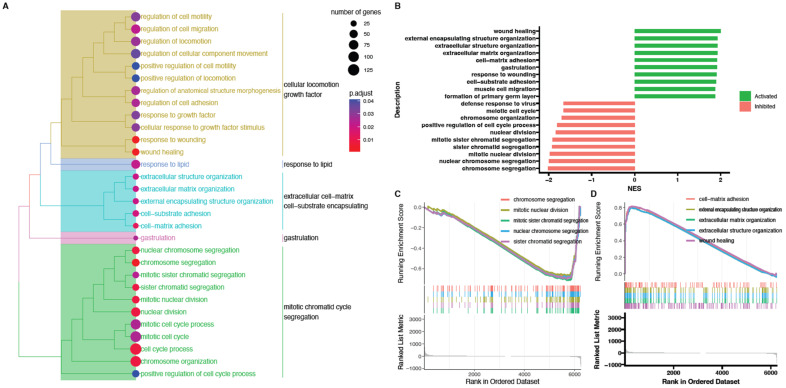
Gene set enrichment analysis (GSEA) cells in 48 h cultivation compared to control (0 h). (**A**) Clusterization of enriched gene sets into common functional clusters. Each cluster is marked with a different color. (**B**) Bar plot with the ten most activated and inhibited gene terms according to the normalized enrichment score (NES) values. (**C**) Detailed enrichment plots for the five most inhibited gene sets showing the profile of the running ES score and positions of genes on the rank-ordered list. (**D**) Detailed enrichment plots for the five most activated gene sets showing the profile of the running ES score and positions of genes on the rank-ordered list.

**Figure 9:**
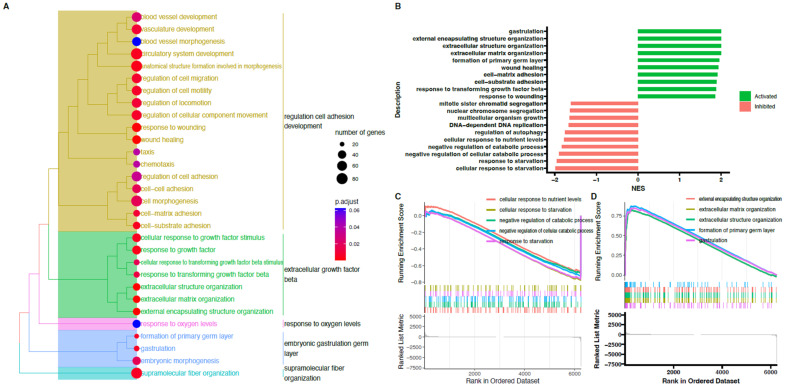
Gene set enrichment analysis (GSEA) cells in 96 h cultivation compared to control (0 h). (**A**) Clusterization of enriched gene sets into common functional clusters. Each cluster is marked with a different color. (**B**) Bar plot with the ten most activated and inhibited gene terms according to the normalized enrichment score (NES) values. (**C**) Detailed enrichment plots for the five most inhibited gene sets showing the profile of the running ES score and positions of genes on the rank-ordered list. (**D**) Detailed enrichment plots for the five most activated gene sets showing the profile of the running ES score and positions of genes on the rank-ordered list.

**Figure 10:**
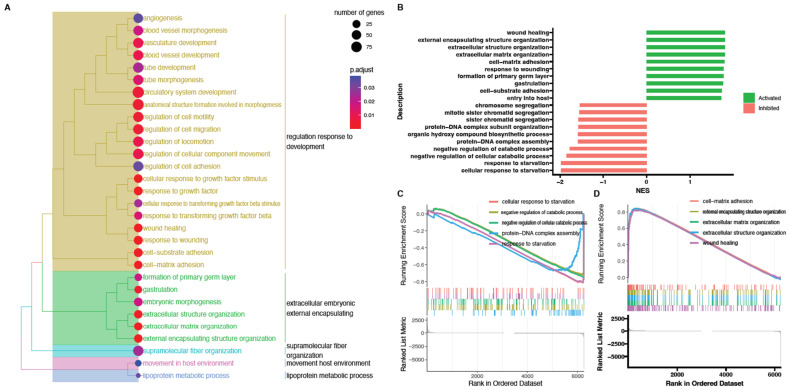
Gene set enrichment analysis (GSEA) cells in 144 h cultivation compared to control (0 h). (**A**) Clusterization of enriched gene sets into common functional clusters. Each cluster is marked with a different color. (**B**) Bar plot with the ten most activated and inhibited gene terms according to the normalized enrichment score (NES) values. (**C**) Detailed enrichment plots for the five most inhibited gene sets showing the profile of the running ES score and positions of genes on the rank-ordered list. (**D**) Detailed enrichment plots for the five most activated gene sets showing the profile of the running ES score and positions of genes on the rank-ordered list.

The strongest enriched term in the comparison between 48 h, 144 h, and 0 h referred to “wound healing.” Meanwhile, the strongest enriched term in the comparison between 96 h and 0 h referred to “gastrulation.” This means that the expression of those terms was higher in the analyzed groups in comparison to controls. Detailed results of this analysis are presented in Figure 8, Figure 9 and Figure 10. Despite a different methodological approach, the GSEA analysis presented relatively similar groups, as shown in the analysis of ontological groups by DAVID. This group’s enriched terms strictly related to the cell cycle pathway, such as wound healing, extracellular matrix organization, and cell-matrix adhesion. 

**Figure 11:**
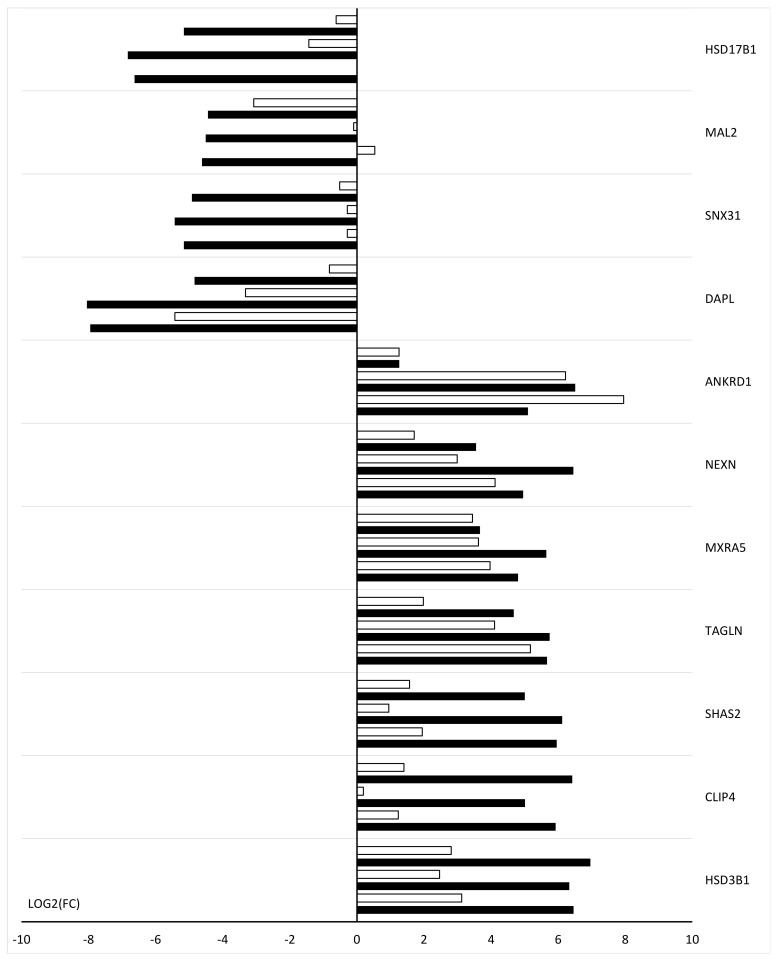
Bar graph showing the microarray validation results obtained by RT-qPCR. The black bar indicates the results of microarray expressions; the white bar indicates the results of RT-qPCR.

Quantitative RT-qPCR was used to validate the results from the microarray expression. Results for 11 selected genes are presented as a bar graph (Figure 11). The differences in gene expression shown in Figure 11 are due to the greater sensitivity of RT-qPCR than microarray expression methods. 

The analyses focused on cellular processes, such as the migration, adhesion, and proliferation of granulosa cells. Extracellular vesicles, mainly exosomes, which represent a form of intercellular signaling based on exocytary release, have been shown to play an important role in these processes. Interestingly, increased expression of genes belonging to the “vesicle—mediated transport” ontological group was demonstrated, indicating an important role for this type of intercellular signaling in cultured granulosa cells.

## 3. Discussion

The granulosa cells’ function in steroidogenesis, folliculogenesis, and oogenesis requires proper intercellular signaling (both physical and chemical). The ECM, cytoskeleton, transmembrane proteins, and multiple signaling pathways are involved in this signaling. It is worth noting that extracellular vesicles are also important in this context, with their role in cell adhesion, cell-to-cell adhesion, migration, and proliferation [9,10,11,19,20,23,24,25,29]. The demonstrated elevated expression of genes mainly related to the processes of migration, proliferation, and granulosa cell adhesion clearly suggests that exosomes are important in these processes. Exosomal influence on the molecular level is not completely understood. A thorough understanding of these mechanisms and the messengers involved can be used in assisted reproductive techniques (ART) and in the treatment of ovarian disorders, such as PCOS (polycystic ovary syndrome) and POI (premature ovarian insufficiency). In addition, given the elevated expression of genes included in the wound healing ontology group in granulosa cells and the previously demonstrated potential for stemness [30,31], as well as the important role of exosomes in tissue regeneration [32], further research linking these aspects is needed. In this study, eleven genes with increased expression (LIPG, HSD3B1, CLIP4, LOX, ANKRD1, FMOD, SHAS2, TAGLN, ITGA8, MXRA5, and NEXN), ten with decreased expression (DAPL1, HSD17B1, SNX31, FST, NEBL, CXCL10, RGS2, MAL2, IHH, and TRIB2), and selected ontology groups were chosen for further analysis.

Cells are the basic building blocks of living organisms, which require numerous interactions among themselves and between the cell and the extracellular environment to function properly. For this purpose, cells exhibit adhesion, which is carried out by various components that build the cell, including cadherins, integrins (cell adhesion molecules—CAM), and the cytoskeleton. Proper communication requires continuous changes in cell adhesion, thereby remodeling the structures involved. These connections show varying degrees of complexity depending on the tissue the cell type builds [33]. An important element in the aspect of intercellular signaling and also in granulosa cells is the extracellular matrix. The combination of the ECM with integrins (transmembrane proteins) allows the transmission of signals. In addition to elevated expression of the ECM-associated genes ITGA2 and ITGB3 in porcine GCs [9], the present study also showed significant upregulation of ITGA8 gene expression. This integrin (ITGA8) has so far been described in bovine cumulus cells, where it is responsible for integrin-mediated cell adhesion [34]. Expression of this gene is also significantly modified by progesterone, which has been shown in the oviduct [35] and may be equally important in the ovary. In reference to previous results [9,34] and the elevated expression of the integrin-mediated signaling pathway ontology group in our research, the role of integrins in cell signaling within the ovarian follicle is highlighted. 

It is noteworthy that in the context of intercellular signaling [36] and cell adhesion [18], an important role has recently been demonstrated for extracellular vesicles, including exosomes. The ECM plays an important role in the transport of extracellular vesicles, which, depending on the degree of stress (resulting from its composition), affects the diffusion of EVs [37]. Exosomes affect target cells through direct contact with extracellular receptors, or, after binding to the cell membrane, can be uptaken by clathrin-dependent endocytosis [38]. After fusion with the cell membrane, exosomes release the transferred type of molecule directly into the cytosol [39] or influence the recipient cell through the activation of signaling pathways [40]. A potential second pathway based on caveolin has also been demonstrated [41]. However, elevated expression of the CAV1 gene has been shown to negatively affect exosome uptake [42]. This is related to the effect of CAV1, which inhibits the ERK1/2 signaling pathway; this signaling pathway showed elevated expression in our research [42]. Interestingly, exosomes are involved in regulating the composition of the extracellular matrix [43,44], thereby affecting the cellular processes mediated by the ECM. The action of exosomes is not only limited to constituting the structural components of the ECM, but also stimulates cells to release enzymes responsible for ECM remodeling (matrix metalloproteases-MMPs) [43]. It is worth adding that the upregulation of the CAV-1 gene, which is responsible for ECM remodeling by participating in exosome formation [45], was presented in our previous studies [9]. As nanoparticles with biological activity, exosomes have a wide range of functions in the mammalian tissues. Due to their characteristics, they are being carefully studied for use as drug transporters and molecular markers of diseases [46]. 

ECM composition and cell adhesion are influenced by the Matrix-remodeling associated (MXRA) protein family [47] and also by fibromodulin (FMOD) [34], which are the genes that showed increased expression (Figure 4 and Figure 5). The composition of the ECM influences the microenvironment of cells and thus is also associated with pathological conditions. Elevated MXRA5 gene expression has been demonstrated in pancreatic cancer [48]. The protein encoded by the LOX gene, Cu-dependent lysyl oxidase (LOX), also has an important effect on ECM remodeling and was upregulated. This protein is involved in a number of signaling pathways, e.g., EGFR, PDGF, VEGF, TGF-β, MAPK, and FAK/AKT [49]. LOX has been shown to interact with the cytoskeleton, and its expression in the nucleus has been demonstrated, suggesting activity in cell division [50,51]. In addition, LOX expression is regulated by integrin–collagen fusion, confirming its role in mechanotransduction [52]. LOX showed a multiplicity of functions in granulosa cells, including effects on signaling pathways MAPK, ERK, and FAK [49], which are important for the function of GCs [9]. And, given the involvement of the LOX gene in the differentiation of pluripotent cells into osteoblasts [53] in relation to the stemness potential of GCs [30,31], this gene requires further careful study. LOX also shows a role in steroidogenesis within rat ovaries [54] and in PCOS [55]. However, it may be an important marker for processes related to reproduction and cell differentiation in GCs. Expression of the LIPG (EL endothelial lipase) gene has not been described in pig granulosa cells. This lipase regulates lipoproteins’ metabolism [56], which, as sources of cholesterol [57], are important for mitochondria-mediated steroidogenesis [58]. Exosomes are also involved in the metabolism of lipids, including cholesterol [59]. LIPG expression is affected by IL-1β [60] similarly to LOX [49], although it is also affected by sex hormones [61]. This indicates that the genes of interest may be crucial for steroidogenesis in porcine granulosa cells. Upregulated 3β-hydroxysteroid dehydrogenase 1 (HSD3B1) plays an important role in steroidogenesis [62]; its expression is dependent on imidacloprid [62] and estrogen [63].

Another ontological group showing increased expression is the repression of apoptosis. The negative regulation of apoptosis in the cancer cells described is associated with increased expression of the ANKRD1 gene. The expression of ANKRD1 was upregulated. The process of natural cell death is also regulated by exosomes through their effect on TNF related apoptosis inducing ligand (TRAIL) [64]. The ANKRD1 gene shows a positive effect on the differentiation of hMSCs into adipocytes and a negative effect on osteoblastocytes [65]. In addition, this gene regulates cell sensitivity to cisplatin, thereby affecting ER stress-induced apoptosis (caused by hypoxia) [66]. TAGLN (transgelin), like the ANKRD1 gene, showed upregulation and is associated with the differentiation of hMSCs into osteoblasts and adipocytes [67]. In view of the potential of GCs to differentiate into other cell types, they may provide a basis for further research in this direction.

Genes belonging to the response to hypoxia ontology group showed significant upregulation in our study. This process in the aspect of the reproductive system, especially the ovarian microenvironment, is very important [68]. It is responsible for maintaining the proper oxygen concentration necessary for folliculogenesis and ovulation [69]. The response to conditions of reduced oxygen concentration involves the release of hypoxia-inducible factors (HIFs) [68]. The reduced oxygen concentration condition also affects the exosomes that are released [21], particularly in the case of exosomes secreted in the tumor microenvironment (TME) [70]. Additionally, changes in exosomes have been described in the context of hypoxia-maintained human umbilical vein endothelial cells (HUVECs), which were later used in regenerative medicine [71]. Hypoxia is linked to the process of angiogenesis as an element very important for the formation of the corpus luteum [72]. The genes encoding proteins involved in the process of angiogenesis showed upregulation. Angiogenesis within the ovarian follicle is very important, and its improper regulation can be associated with various disorders, such as PCOS and POI [73]. Each ovarian follicle undergoing folliculogenesis manifests a temporary, individual vascularization pattern. It has been shown that inhibition of angiogenesis within the ovary slows the depletion of the ovarian follicle pool and can be used to treat POI [74]. Exosomes have been shown to promote angiogenesis through the suppression of HIK-1 expression [75] as well as by microRNA-92a-3p [76]. 

Another ontological group showing increased expression is wound healing. This process strictly depends on the blood supply to the tissues undergoing healing. The healing process is also closely related to exosomes [77,78,79], which significantly influence its course (probably also by influencing angiogenesis [75,76]), and they are important for their application in clinical practice [80]. The involvement of previously described ANKRD1 and LOX genes, whose expression was upregulated, was demonstrated to display an important role in wound healing. ANKRD1 affects the interaction of fibroblasts with collagen fibers [81]. LOX, on the other hand, is involved in ECM remodeling during new tissue reconstruction [82].

Cell migration is very important for many processes related to development, embryogenesis, immune response, and wound healing, among others. The current study demonstrated increased expression of genes belonging to the cell migration ontology group. SHAS2 (swine hyaluronic acid synthase 2) was upregulated in the present study; it is mainly responsible for the cumulus expansion process (one of the LH-mediated ovulatory processes), and inhibition of its expression leads to reduced migration of granulosa cells [83]. This gene is also responsible for the synthesis of hyaluronyan, the main component of the ECM [84]. In addition, hyaluronic acid is an important component affecting exosomes with regard to their bone-regenerative capacity [85]. SHAS2 has been described in pig CCs [86]. Additionally, cell viability and migration depend on the expression of the CLIP4 gene (upregulated (Figure 3 and Figure 5)), whose knockdown causes a significant decrease in cell viability [87]. EVs, including exosomes, also have an impact on migration [88,89]. They play a key role in migration, conducting it in an autocrine and paracrine way [19]. Exosomes stimulate extracellular signaling receptors, and their deposition near the cell membrane is required to initiate the migration process [90]. In addition, interactions between exosomes and the ECM via integrins, among other things, show the importance of connecting the cell to the extracellular environment [91]. 

Downregulation of the HSD17B1 (hydroxysteroid 17-beta dehydrogenase 1) gene results in a decrease in estrogen because the enzyme is responsible for the last step of steroidogenesis in porcine granulosa cells. This process is further regulated positively by the p53 protein and negatively by FoxA2 [92]. Changes in sex hormone levels in relation to PCOS have also been shown to be caused by changes in HSD17B1 gene expression in follicular fluid (FF) exosomes [93]. A negative effect of dioxin on the expression of the HSD17B1 gene has been described, thereby causing the inhibition of steroidogenesis [94]. In addition, this gene has been identified as a marker of steroidogenesis in ovine granulosa cells, thus affecting fecundity in this species [95]. A downregulation of FST (follistatin) was revealed in the current study, which could positively affect porcine GCs’ proliferation and estrogen secretion [96]. FST also affects the TGF-β signaling pathway, which is closely responsible for ovarian follicle development [97] and the survival rate of follicles [98]. Expression of the CXCL10 (C-X-C motif chemokine ligand 10) gene exhibited reduced expression, but it has no effect on steroidogenesis within luteinized ovarian granulosa cells [99]. However, CXCL10 has been shown to affect the production of COL1A1 and COL1A2, which, as a component of the ECM, can affect fibrosis within the ovary, leading to POI [99].

## 4. Materials and Methods

### 4.1. Animals

Ovaries were collected post-slaughter from 40 sexually mature gilts. Animals slaughtered in a commercial slaughterhouse were kept under similar breeding conditions on registered farms. At slaughter, the animals had reached an average weight of 98 kg and an age of about 6 months (+/−10 days).

### 4.2. Collection of Porcine Ovarian Granulosa Cells

The research material was transported to the laboratory at 38 °C in 0.9% NaCl within 30 min of harvesting. In the laboratory, ovaries isolated from the reproductive organs were placed in PBS (phosphate-buffered saline) solution supplemented with fetal bovine serum (FBS; Sigma-Aldrich Co., St. Louis, MO, USA). Follicular fluid (FF) was then aspirated from individual pre-ovulatory ovarian follicles larger than 5 mm in diameter using a 5 mL syringe and a 20 G needle. The fluid thus extracted was deposited into a sterile Petri dish, and then cumulus–oocyte complexes (COCs) were recovered for rejection. The extracted vesicular fluid after COCs rejection was filtered through sterile nylon cell screens with a mesh diameter of 40 µm (Biologix Group, Shandong, China) to eliminate tissue debris and larger cell aggregates, including erythrocytes and epithelial cells. The resulting suspension was centrifuged at room temperature for 10 min at 200× *g* to divide the solution into fractions. After discarding the supernatant, the GCs pellet was then suspended in collagenase type I solution (Gibco, Thermo-Fischer Scientific, Waltham, MA, USA) and 1 mg/1 mL DMEM and incubated for 10 min in a 37 °C water bath, followed by centrifugation (under the same conditions as stated above). Granulosa cells were taken from different ovarian follicles to homogenize the sample, and the pellet obtained after centrifugation was used to establish the primary culture. 

### 4.3. In Vitro Primary Culture of Porcine Granulosa Cells

A primary in vitro culture model was used in this study with four time intervals. For microarray expressions, cultures were maintained in two biological replicates for each time interval. For validation by RT-qPCR, cultures were maintained in a triplicate biological sample model for each time interval. Primary cultures were established from GCs in four bottles. Cells were seeded at 3 × 10^6^/culture bottle (25 cm^2^, TPP, Trasadingen, Switzerland). The number of cells and their viability were assessed using an ADAM automatic cell counter (NanoEnTek, Waltham, MA, USA). Only samples with a cell viability above 85% were used for further studies. The culture medium consisted of Dulbecco’s Modified Eagle’s Medium (DMEM, Sigma-Aldrich, Saint Louis, MO, USA), 2% fetal calf serum (FCS) (PAA, Linz, Austria), 10 mg/mL ascorbic acid (Sigma-Aldrich, Saint Louis, MO, USA), 0.05 μM dexamethasone (Sigma-Aldrich, Saint Louis, MO, USA), 200 mM L-glutamine (Invitrogen, Carlsbad, CA, USA), 10 mg/mL gentamicin (Invitrogen, Carlsbad, CA, USA), 10,000 units/mL penicillin (Invitrogen, Carlsbad, CA, USA), and 10,000 μg/mL streptomycin (Invitrogen, Carlsbad, CA, USA). The culture bottles prepared in this way, together with the cells, were maintained at 38.5 °C and 5% CO2. After the cells reached more than 80% confluence, they were detached from the medium with 0.05% trypsin-EDTA (Invitrogen, Carlsbad, CA, USA) and then passaged. Cells in culture were kept until culture termination, and the material was collected at 0 h, 48 h, 96 h, and 144 h. The culture medium was changed every 72 h.

### 4.4. Microarray Expression Analysis and Statistics

The total RNA from porcine granulosa cells was isolated using TRI Reagent (Sigma, St Louis, MO, USA), and an RNeasyMinElute cleanup Kit (Qiagen, Hilden, Germany). The RNA for transcriptome study was collected from two independent replicates for each experimental variant: (1) control—0 h, (2) 48 h, (3) 96 h, and (4) 144 h. Each replicate contained pooled RNA from three independent experiments. The microarray study was performed according to the previously described protocol [100,101].

First, the total RNA (100 ng) from each sample was submitted to a two-step cDNA synthesis reaction, biotin labeling, and fragmentation according to the manufacturer’s instructions (GeneChip^®^ WT Plus Reagent Kit, Affymetrix, Santa Clara, CA, USA). Then, the biotin-labeled fragments of cDNA were hybridized to the Affymetrix^®^ PorGene 1.1 ST Array Strip (45 °C/20 h). Next, the microarrays were stained by the Affymetrix GeneAtlas Fluidics Station of GeneAtlas System. The microarrays were scanned by the Imaging Station of the GeneAtlas System (Affymetrix, Santa Clara, CA, USA). The Affymetrix GeneAtlas Operating System was performed for the analysis of the obtained results. The quality of the gene expression data was confirmed using the software’s quality control criteria. 

All analyses were performed by BioConductor software with the relevant Bioconductor libraries through the statistical R programming language (v4.1.2; R Core Team 2021). For the normalization, background correction, and calculation of the expression values of the analyzed genes, the robust multiarray average (RMA) normalization algorithm implement in the “Affy” library was applied [102]. To show the total number of upregulated and downregulated genes, the principal component analysis (PCA) of the filtered data set was performed and visualized using the “factoextra” library [103]. Next, the DAVID (Database for Annotation, Visualization, and Integrated Discovery) bioinformatics tool was used for functional annotation and clusterization of differentially expressed genes (DEGs) [102,104]. The established cut-off criteria for DEGs was based on the differences in the absolute value from the expression fold change greater than 2. Furthermore, the expressed genes were assigned to relevant GO terms, with the subsequent selection of significantly enriched GO terms using the GO BP DIRECT database. The *p*-values of selected GO terms were corrected using the Benjamini–Hochberg correction [105]. DEGs from each comparison were visualized through hierarchic clustering of differentially expressed genes as a heatmap using the “ComplexHeatmap” library [106]. Genes belonging to the first seven most significantly enriched ontological groups (lowest adjusted *p*-value) were shown on the figures with the expression values of analyzed genes.

GSEA was carried out using the “clusterProfiler” Bioconductor library [107]. The aim of the analysis was to identify the level of depletion or enrichment in GO terms through the calculation of the normalized enrichment score (NES) with the relevant *p*-value. Normalized fold change values from all of the genes were log2 transformed, sorted, and used as an argument for the “gseGO” function. Gene set enrichment was performed with reference to the “biological process” GO category, assuming that the minimum size of each geneSet for analyzing = 50 and *p*-value cut-off = 0.05. Then, hierarchical clustering of enriched terms based on pairwise similarities calculation with Jaccard’s similarity index was performed. The result of the analysis qualified individual GO terms to clusters based on their functional similarity. The obtained clusters were presented as a tree plot. The ten ontology groups with the highest enrichment score (the highest NES value) and the ten groups with the most depleted enrichment score (the lowest NES value) were visualized as a bar chart. Enrichment plots for five of the most enriched and depleted GO terms were also presented.

### 4.5. Real-Time Quantitative Polymerase Chain Reaction (RT-qPCR) Analysis

Total RNA was isolated from GCs at 0 h and after 48 h, 96 h, and 144 h in vitro culture using an RNeasy mini column from Qiagen GmbH (Hilden, Germany). The RNA samples were resuspended in 20 µL of RNase-free water and stored in liquid nitrogen. RNA samples were treated with DNase I and reverse-transcribed (RT) into cDNA. RT-qPCR was conducted in a LightCycler real-time PCR detection system (Roche Diagnostics GmbH, Mannheim, Germany) using SYBR^®^ Green I as a detection dye, and the target cDNA was quantified using the relative quantification method. The relative abundance of analyzed transcripts in each sample was standardized to the internal standard glyceraldehyde-3-phosphate dehydrogenase (GAPDH). For amplification, 2 µL of cDNA solution was added to 18 µL of QuantiTect^®^ SYBR^®^ Green PCR (Master Mix Qiagen GmbH, Hilden, Germany) and primers (Table 1). One RNA sample of each preparation was processed without the RT reaction to provide a negative control for subsequent PCR. Eleven randomly selected genes were chosen to validate the microarray results.

To quantify the specific genes expressed in the GCs, the expression levels of specific mRNAs in each sample were calculated relative to PBGD and ACTB. To ensure the integrity of these results, the additional housekeeping gene, 18S, was used as an internal standard to demonstrate that PBGD and ACTB mRNAs were not differentially regulated in GC groups. The gene for 18S rRNA expression has been identified as an appropriate housekeeping gene for use in quantitative PCR studies. The expression of PBGD, ACTB, and 18S mRNA was measured in cDNA samples from isolated GCs. The statistical significance of the analyzed genes was performed using moderated t-statistics from the empirical Bayes method. The *p*-value was corrected for multiple comparisons using Benjamini and Hochberg’s false discovery rate.

## 5. Conclusions

There appears to be an association between the expression of genes involved in cell adhesion, proliferation, migration, division, and intercellular signaling and EV production and composition in granulosa cells. The literature suggests that the ECM and cytoskeleton are also involved in these signaling pathways of granulosa cells. The exosomes in the microenvironment of granulosa cells affect the composition of the ECM, which is a key element of the ovulation process. ECM is also crucial in the aspect of reproductive disorders, such as PCOS and POI. Therefore, these studies can be used to identify genetic markers of processes, largely based on EVs, that can be used in assisted reproductive techniques (ART), reproductive tract disorders, and regenerative medicine.

## Figures and Tables

**Table 1:** Oligonucleotide sequences of primers used for RT-qPCR analysis.

Gene	Primer Sequence (5′-3′)		Product Size (bp)
HSD17B1	F	GTGTCAGAGGCTTGCTAGGG	200
	R	CAGCACAATCTCAAGGCTGA	
MAL2	F	ATCCTCGTCATGGAAAGGTG	202
	R	TGCCACTCATTCATGGTTGT	
SNX31	F	AGGTGACCTTCCTTGGGACT	222
	R	CCGGAACTTCAATCTGCATT	
DAPL	F	CCTGCTCTGGAGAAGGTCAC	151
	R	GGGCCTAAGGAAAGTTTTGG	
ANKRD1	F	CTGCTTGAGGTGGGGAAGTA	178
	R	GTGTCTCACTGTCTGGGGAA	
NEXN	F	GAAGCAAGGAGAAGCATGGC	151
	R	CCTCCTCTGTTCGTCGTCTT	
MXRA5	F	TGCTGGCACTGTTTTCTCAC	212
	R	TCGGAGAGGATTCATGAGGC	
TAGLN	F	TTAAAGGCCGCTGAGGACTA	233
	R	ATGACATGCTTTCCCTCCTG	
SHAS2	F	ATCGCGGCCTATCAAGAAGA	204
	R	GCCCTTTTCGTGGAAGTTGT	
CLIP4	F	CCCTTAGAAATGGCCGATGC	162
	R	ATCTCCCAACTTCAGGCCAA	
HSD3B1	F	TCCACACCAGCAGCATAGAG	245
	R	CATGTGGGCAAAGATGAATG	

## Data Availability

Not applicable.
